# Deep Conversion of Carbon Monoxide to Hydrogen and Formation of Acetate by the Anaerobic Thermophile *Carboxydothermus hydrogenoformans*

**DOI:** 10.1155/2011/641582

**Published:** 2011-07-07

**Authors:** Anne M. Henstra, Alfons J. M. Stams

**Affiliations:** ^1^Centre for Biomolecular Sciences, University of Nottingham, University Park, NG7 2RD Nottingham, UK; ^2^Laboratory of Microbiology, Wageningen University, Dreijenplein 10, 6703 HB Wageningen, The Netherlands

## Abstract

*Carboxydothermus hydrogenoformans* is a thermophilic strictly anaerobic bacterium that catalyses the water gas shift reaction, the conversion of carbon monoxide with water to molecular hydrogen and carbon dioxide. The thermodynamically favorable growth temperature, compared to existing industrial catalytic processes, makes this organism an interesting alternative for production of cheap hydrogen gas suitable to fuel CO-sensitive fuel cells in a future hydrogen economy, provided sufficiently low levels of CO are reached. Here we study CO conversion and final CO levels in cultures of *C. hydrogenoformans* grown in batch cultures that were started with a 100% CO gas phase with and without removal of formed CO_2_. Final CO levels were 117 ppm without CO_2_ removal and below 2 ppm with CO_2_ removal. The Gibbs free energy change calculated with measured end concentrations and the detection of acetate suggest that *C. hydrogenoformans* shifted from a hydrogenogenic to an acetogenic metabolism.

## 1. Introduction

*Carboxydothermus hydrogenoformans* is a strictly anaerobic carboxydotrophic hydrogenogenic thermophilic bacterium [1] that conserves energy for growth by performing the water gas shift reaction, the conversion of carbon monoxide with water to hydrogen and carbon dioxide (reaction (1)). The standard Gibbs free energy change of the reaction per mol of CO is relatively small, −20 kJ mol^−1^. Production of acetate from CO (reaction (2)) or from H_2_ and CO_2_ (reaction (3)) is not reported for *C. hydrogenoformans*, despite the presence of the required genes in the genome of *C. hydrogenoformans* [2]

(1) $$\begin{array}{c} \text{CO}+{\text{H}}_{2}\text{O}\to {\text{CO}}_{2}+{\text{H}}_{2} \end{array}$$

(2) $$\begin{array}{c} 4\text{CO}+2{\text{H}}_{2}\text{O}\to 2{\text{CO}}_{2}+{\text{CH}}_{3}\text{COOH} \end{array}$$

(3) $$\begin{array}{c} 4{\text{H}}_{2}+2{\text{CO}}_{2}\to {\text{CH}}_{3}\text{COOH} \end{array}$$

The water gas shift reaction is applied in industry for production of relative cheap hydrogen gas from synthesis gas. Synthesis gas is a mixture of mainly H_2_, CO, and CO_2_ which is produced by partial oxidation or autothermal reforming of hydrocarbon-rich sources such as fossil fuels, domestic and agricultural wastes, and other biomass sources [3–5]. Water-gas shift catalysis is applied in successive high and low temperature steps at 400 and 200°C to convert the bulk of CO to final concentrations of not below 1000 ppm. Low-temperature fuel cells are sensitive to CO and require CO levels of below 10 ppm or of a few hundred ppm for novel types that apply improved membranes [6]. Hydrogen gas derived from synthesis gas, while relatively cheap, is therefore not suitable for these types of fuel cells. Highly desired is a water-gas shift process capable of removing CO from synthesis gas to levels below 10 ppm in a single step.

Synthesis gas is also of interest as source for tentative biotechnological processes. Several anaerobic microorganisms are known that can convert the synthesis gas constituents (CO and H_2_ + CO_2_) into valuable products, for example, methane, ethanol, butanol, and polyhydroxyalkanoates, in what is called synthesis gas fermentation [7, 8]. More microorganisms exist that produce these compounds with only H_2_ + CO_2_, but these are often sensitive to CO. 

Biotechnological application of microorganisms like *C. hydrogenoformans* to produce a hydrogen gas with minimal CO is thus interesting. They bring the advantage that they operate at lower temperatures than existing water-gas shift catalyst, which is thermodynamically favorable for the removal of CO. Here, we describe the results of a study on CO conversion by and final CO thresholds of *C. hydrogenoformans* metabolism in batch culture. Removal of CO_2_ is studied as it is a means to acquire lower final CO thresholds. The effect on metabolism in relation to minimal free energy change is discussed.

## 2. Materials and Methods

*C. hydrogenoformans* (DSM6008) was obtained from the German Culture Collection (DSMZ, Braunschweig) and cultured in 585 mL serum-stoppered bottles in a medium buffered with 200 mL MOPS [9] and a 100% CO gas phase at 65°C and 200 rpm (1^*″*^ stroke Innova 44 incubator, New Brunswick Scientific). Bottles contained a glass reaction tube with either 5 mL water as control or 5 mL 10 M NaOH to serve as CO_2_ trap. CO, H_2_, CO_2_, acetate, and growth of *C. hydrogenoformans* were analysed as described [9]. Gas and liquid samples were taken with intervals of 180 minutes until 15.5 h. Trace levels of CO (*P*_CO_ < 800 Pa) were analysed on a GC2010 fitted with MTN-1 methanizer (Shimadzu, Japan) and FID by injecting gas samples of 100 *μ*L with a glass gastight syringe that were allowed to equilibrate with atmospheric pressure just before injection into the GC. The Gibbs free energy changes were calculated with observed gas partial pressures and tabulated data for 70°C [10]. Partial pressures of CO_2_ and H_2_ at 15.5 h were used for calculation of Δ*G* of later samples where only trace CO was measured, unless stated otherwise.

## 3. Results and Discussion

**Figure 1:**
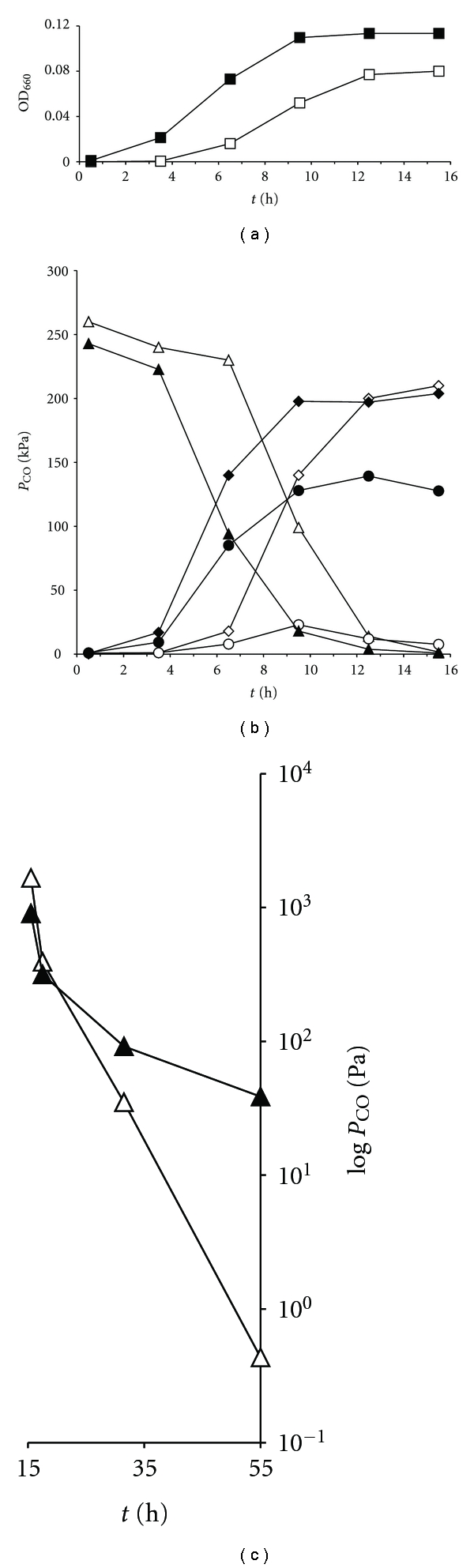
Final CO levels of the hydrogenogenic CO metabolism of *C. hydrogenoformans* was studied batch cultures, with CO_2_ trap and without CO_2_ trap (open versus closed symbols, resp.), in triplicate. Optical density of cultures ((a), square symbols), and partial pressures of CO ((b), triangles), H_2_ (diamonds), and CO_2_ (circles) were monitored over time and plotted on a linear axis. Trace levels of CO (c) were plotted on a logarithmic axis from 15.5 h onward.

The metabolic capacity of *C. hydrogenoformans* cultures to achieve low CO concentrations by CO conversion to H_2_ and the effect of CO_2_ removal were studied. *C. hydrogenoformans* was grown in batch cultures with a CO gas phase. Incubations were done with and without a CO_2_ trap. The optical density and CO, H_2_, and CO_2_ concentrations were measured over time (Figure 1). The final pH and acetate concentrations were also measured. With measured concentrations, the Gibbs free energy changes for reaction (1) and (2) were calculated (Table 1).

The cultures with CO_2_ trap showed very similar CO uptake rates and H_2_ production rates compared to cultures without trap, however, with a clearly longer lag phase and lower final OD than cultures without trap. Likely, the removal of CO_2_ affects growth, as it is an intermediate in the carbon assimilation by *C. hydrogenoformans* through the acetyl-CoA pathway. Additionally, the CO_2_ trap, which is an alkaline solution, could trap the acid gas, H_2_S, which serves as source of sulfur and the low redox potential needed for growth of *C. hydrogenoformans*. Once CO conversion in cultures with CO_2_ trap reached comparable rates to cultures without trap, CO_2_ started to accumulate until most CO was consumed. Thereafter CO_2_ dropped to end point concentrations below the detection limits (<350 kPa). Final CO levels in cultures without CO_2_ trap were 117 ppm, while in cultures with CO_2_ trap the CO levels had dropped below the detection limit of 2 ppm.

With measured concentrations, using detection limits where concentrations could not be measured, the Gibbs free energy change was calculated for reactions (1) and (2) (Table 1). For reaction (1), the conversion of CO with water to CO_2_ and H_2_, Δ*G* were +1.4 and −3 kJ/mol CO for cultures without and with CO_2_ trap, respectively. These values are closer to thermodynamic equilibrium (Δ*G* = 0) than can be expected based on minimal biological energy quantum theory that takes the Gibbs free energy to translocate one proton over the cytoplasmic membrane as minimum, which was assumed −20 kJ mol^−1^ [11], even when a degree of variability is allowed for the minimal biological energy quantum [12]. Instead of reaction (1), the conversion of CO to H_2_, another metabolic reaction that causes the removal of CO in later stages of culture. Acetate was detected in cultures without CO_2_ trap, which suggests *C. hydrogenoformans* shifted from reaction (1) to reaction (2) during cultivation. The Δ*G* for reaction (2) with average concentration of 3.8 mM acetate was −18 kJ mol^−1^ for cultures without CO_2_ trap, which is much closer to theoretical −20 kJ mol^−1^ of the minimal biological energy quantum. However, in cultures with CO_2_ trap, acetate was not detected. The detection limit for acetate was 0.2 mM. It is possible that enough CO or CO_2_ was not available to produce more acetate than the detection limit in cultures with CO_2_ trap. With 0.2 mM acetate, a Δ*G*_R2_ of −16 kJ mol^−1^ CO was calculated which is more in accordance with the minimal biological energy quantum.

## 4. Conclusion

Batch cultivation of *C. hydrogenoformans* with CO resulted in final CO concentrations of 117 ppm. With removal of CO_2_ during cultivation, even lower CO concentrations of below 2 ppm were reached. The low CO levels in the produced hydrogen-rich gas make the gas suitable for application in CO-sensitive processes. Low-temperature fuel cells require CO to be present below 10 ppm [8]. Improved fuel cell membranes allow several hundred ppm [13, 14]. While the bulk of CO was converted to H_2_ by *C. hydrogenoformans*, it is likely that metabolism shifted to production of acetate from CO in final stages of incubation. Production of acetate by *C. hydrogenoformans* was not shown before. To apply such a biological catalyst, further research is needed on kinetic aspects, mainly relating to mass transfer at the gas-liquid-microbe interfaces.

## Figures and Tables

**Table 1:** Observed partial pressures and the calculated Gibbs free energy change of CO conversion by *C. hydrogenoformans* in batch culture.

Culture	*t* (h)	H_2_ (Pa)	CO (Pa)	CO_2_ (Pa)	Acetate (mM)	Δ*G*_R1_^(a)^	Δ*G*_R2_^(a)^
−CO_2_ trap	0.5	6.0 · 10^2^	2.4 · 10^5^	1.1 · 10^3^	0.2 ^ (b)^	−53	−52
	9.5	2.0 · 10^5^	1.8 · 10^4^	1.3 · 10^5^		−16	
	55	2.0 · 10^5^	3.9 · 10^1^	1.3 · 10^5^	3.8	+1	−18

+CO_2_ trap	0.5	2.2 · 10^2^	2.6 · 10^5^	8.4 · 10^2^	0.2	−57	−52
	12.5	2.0 · 10^5^	1.4 · 10^4^	1.2 · 10^4^		−22	
	55	2.1 · 10^5^	$\underline{0.4\cdot 10^{0}}$	$\underline{3.5\cdot 10^{2}}$	0.2	−3	−16

^(a)^Δ*G*_R1_ and Δ*G*_R2_: Gibbs free energy change (kJ/mol CO) for reaction (1) and reaction (2), respectively, at 70°C [10].

^(b)^Underlined: detection limits that were used in calculation of free energy.
